# Stabilization of Hfq-mediated translational repression by the co-repressor Crc in *Pseudomonas aeruginosa*

**DOI:** 10.1093/nar/gkab510

**Published:** 2021-06-17

**Authors:** Ewelina M Malecka, Flavia Bassani, Tom Dendooven, Elisabeth Sonnleitner, Marlena Rozner, Tanino G Albanese, Armin Resch, Ben Luisi, Sarah Woodson, Udo Bläsi

**Affiliations:** Department of Biophysics, 3400 N. Charles Street, Johns Hopkins University, Baltimore, MD-21218, USA; Department of Microbiology, Immunobiology and Genetics, Max Perutz Labs, University of Vienna, Vienna Biocenter (VBC), Dr. Bohrgasse 9/4, 1030 Vienna, Austria; Department of Biochemistry, University of Cambridge, Cambridge CB2 1GA, UK; Department of Microbiology, Immunobiology and Genetics, Max Perutz Labs, University of Vienna, Vienna Biocenter (VBC), Dr. Bohrgasse 9/4, 1030 Vienna, Austria; Department of Microbiology, Immunobiology and Genetics, Max Perutz Labs, University of Vienna, Vienna Biocenter (VBC), Dr. Bohrgasse 9/4, 1030 Vienna, Austria; Department of Microbiology, Immunobiology and Genetics, Max Perutz Labs, University of Vienna, Vienna Biocenter (VBC), Dr. Bohrgasse 9/4, 1030 Vienna, Austria; Department of Microbiology, Immunobiology and Genetics, Max Perutz Labs, University of Vienna, Vienna Biocenter (VBC), Dr. Bohrgasse 9/4, 1030 Vienna, Austria; Department of Biochemistry, University of Cambridge, Cambridge CB2 1GA, UK; Department of Biophysics, 3400 N. Charles Street, Johns Hopkins University, Baltimore, MD-21218, USA; Department of Microbiology, Immunobiology and Genetics, Max Perutz Labs, University of Vienna, Vienna Biocenter (VBC), Dr. Bohrgasse 9/4, 1030 Vienna, Austria

## Abstract

In *Pseudomonas aeruginosa* the RNA chaperone Hfq and the *c*atabolite *r*epression *c*ontrol protein (Crc) govern translation of numerous transcripts during carbon catabolite repression. Here, Crc was shown to enhance Hfq-mediated translational repression of several mRNAs. We have developed a single-molecule fluorescence assay to quantitatively assess the cooperation of Hfq and Crc to form a repressive complex on a RNA, encompassing the translation initiation region and the proximal coding sequence of the *P. aeruginosa amiE* gene. The presence of Crc did not change the *amiE* RNA-Hfq interaction lifetimes, whereas it changed the equilibrium towards more stable repressive complexes. This observation is in accord with Cryo-EM analyses, which showed an increased compactness of the repressive Hfq/Crc/RNA assemblies. These biophysical studies revealed how Crc protein kinetically stabilizes Hfq/RNA complexes, and how the two proteins together fold a large segment of the mRNA into a more compact translationally repressive structure. In fact, the presence of Crc resulted in stronger translational repression *in vitro* and in a significantly reduced half-life of the target *amiE* mRNA *in vivo*. Although Hfq is well-known to act with small regulatory RNAs, this study shows how Hfq can collaborate with another protein to down-regulate translation of mRNAs that become targets for the degradative machinery.

## INTRODUCTION

The opportunistic human pathogen *P. aeruginosa* (*Pae*) features two extensive post-transcriptional networks that depend on Hfq (1,2) and the CsrA-like Rsm proteins (3,4). Hfq-dependent regulation controls virulence traits (5–7), including the susceptibility to clinically relevant antibiotics (8–10). As in Enterobacteriaceae (11), Hfq was shown to exert these functions in *Pae* by assisting riboregulation by small RNAs (sRNAs; 10,12–14) as well as by directly acting as a translational repressor on target mRNAs (2,7). The CsrA/RsmA family of dimeric translational repressors control virulence gene expression in pathogenic Bacteria (3,15) including *Pae* (16). Members of this family recognize a GGA motif usually present in the Shine and Dalgarno sequence of target mRNAs that is exposed in the loop of a hairpin (17,18).

In Bacteria, the uptake and utilization of carbon compounds is controlled in a hierarchical manner by a mechanism known as carbon catabolite repression (CCR). Whereas in Enterobacteriaceae and Firmicutes CCR is predominantly controlled at the level of transcription (19), in *Pseudomonas* spp. CCR operates at the post-transcriptional level (20). In *Pae* Hfq was shown to act as a translational repressor of uptake and degradative functions required for utilization of carbon and nitrogen sources other than the preferred one (2). Several studies revealed that Hfq forms a repressive complex together with the *c*atabolite *r*epression *c*ontrol protein Crc on target mRNAs, e.g. *amiE* mRNA encoding an aliphatic amidase (21,22). As Crc was shown to promote Hfq-mediated translational repression of several mRNAs (2), it can be regarded as a translational co-repressor. Moreover, a recent ChIP-seq study and a combined RNA-seq/proteomics approach identified 100- (1) and 244 mRNAs (23), respectively, to be regulated by Hfq and Crc. Cryo-EM studies showed that a segment encompassing the ribosome binding site of *amiE* mRNA is sandwiched between Hfq and Crc, thus rationalizing the auxiliary function of Crc in Hfq-mediated translational repression by obstructing initiating ribosomes (24).

The response to different carbon sources is mediated through different levels of the regulatory RNA CrcZ (25,26). As CrcZ displays several Hfq binding motifs, it sequesters Hfq when CCR is alleviated (2,22), which in turn directs metabolism to use less preferred carbon and nitrogen sources. CrcZ expression is under control of the alternative sigma factor RpoN and the two component signalling system CbrA/B (25,27). As CrcZ acts as a decoy for Hfq, it interferes not only with direct translational repression by Hfq/Crc (2) but also with Hfq-mediated riboregulation by sRNAs (13), with impact on biofilm formation (28) and antibiotic susceptibility (8,9).

In isolation, the Crc protein has no intrinsic affinity for either RNA (29) or Hfq (22). However, it can cooperate with Hfq to assemble a complex on RNAs that contain (AAN)_n_ or (ARN)_n_ repeats, in which A is an adenine, R is a purine (A/G) and N is any nucleotide, which align to engage the six tripartite binding pockets on the distal face of Hfq (22,30–32). Although the presence of Crc did not significantly enhance the affinity of Hfq for an *amiE* derived RNA octadecamer, encompassing five consecutive AAN triplets followed by one ARN triplet (*amiE*_6ARN_), the Hfq/Crc/*amiE*_6ARN_ assembly displayed an increased stability when compared with the Hfq/*amiE*_6ARN_ complex alone (22). Structural studies on Hfq/Crc/*amiE*_6ARN_ complex supported and explained these findings. The assembly was formed by two stacked Hfq hexamers with each bound to one *amiE*_6ARN_ motif and two interconnecting Crc protomers, predominantly contacting the RNA molecules bound to the Hfq hexamers (24).

**Figure 1.**
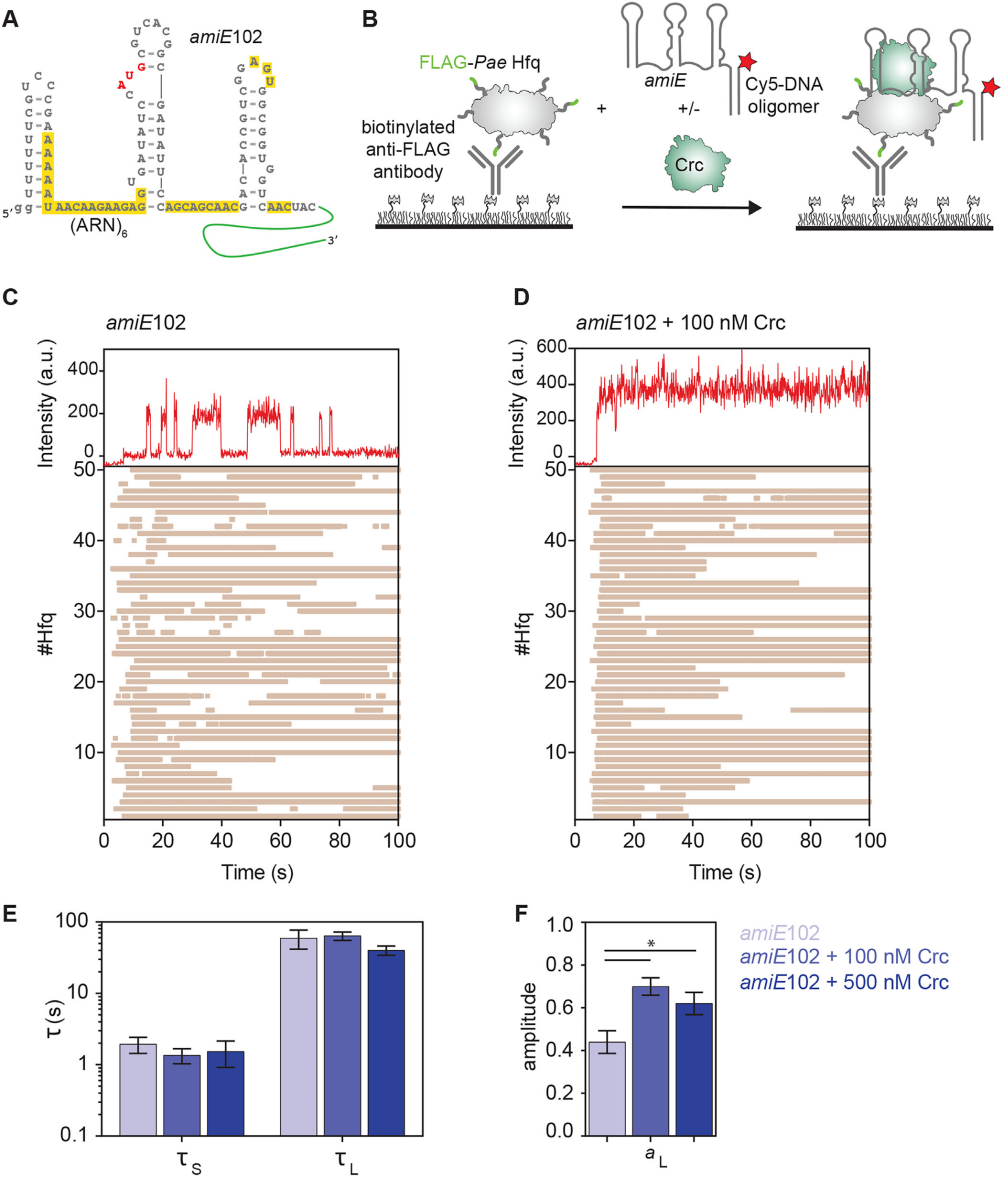
Crc stabilizes Hfq/RNA complexes. (**A**) Predicted secondary structure (54) of *amiE*102 RNA. The two G nucleotides (gg) at the 5′end were added to facilitate *in vitro* transcription. The *amiE*_6ARN_ motif overlapping with the Shine and Dalgarno sequence, which can fully occupy the six tripartite A(A)RN binding pockets on the distal side of Hfq (24), and putative AA(R)N motifs downstream of the AUG are highlighted in yellow. (**B**) Schematic of the single-molecule assay using immobilized Hfq* and Cy5-labeled *amiE*102 RNA. The sequence of the extension present in *amiE*102 RNA is indicated in green. (C–F) Representative binding events for single Hfq* hexamers in the absence or presence of Crc. Top panels, fluorescence intensity upon Cy5 excitation with 0.1 s frames. Bottom panels, rastergrams depicting Hfq*/RNA or Hfq/Crc/RNA interactions. 50 randomly selected traces are shown. Each horizontal bar represents an individual binding event. (**C**) Hfq*/*amiE*102 binding; (**D**) Hfq*/*amiE*102 binding in the presence of 100 nM Crc; (E, F). The presence of Crc increased the population of long-lived RNA-Hfq* complexes. Comparison of the lifetimes (τ_s (short)_ and τ_L (long)_; **E**) and amplitudes for the long lifetime (a_L_; **F**) from maximum likelihood analysis of the dwell times. Details are shown in Supplementary Figure S2 and Supplementary Table S1. The error bars represent the s.d. of the mean determined from bootstrap analysis. Details of the number of observations and fit parameters can be found in Supplementary Figure S2 and Supplementary Table S1. The p values were calculated using an unpaired *t*-test. * indicates *P* < 0.05.

Here, we have studied Hfq/Crc complexes formed on a longer *amiE* mRNA sub-sequence (*amiE*102 RNA; Figure 1A), encompassing the translation initiation region and the proximal coding sequence. Single-molecule co-localization experiments revealed that the presence of Crc did not change the *amiE*102 RNA-Hfq interaction lifetimes. However, Crc increased the probability of forming stable complexes, which is supported by Cryo-EM studies, showing that the presence of Crc increases the compactness of the repressive complexes. In *vitro* translation assays corroborate these findings as the presence of Crc resulted in stronger translational repression, which can be reconciled with the significantly reduced stability of *amiE* mRNA in *Pae* strain PAO1 when compared with PAO1*Δcrc*.

## MATERIALS AND METHODS

### Strains and Plasmids

The strains and plasmids used in these studies are indicated throughout the text and newly constructed plasmids are described in Supplementary Materials and Methods.

### Protein purification

The *P. aeruginosa* Hfq/Hfq_S65C_-Flag heterooligomer (Hfq*) used in the single-molecule assays was produced in *E. coli* strain JW4130F′Δ*hfq*(pKEHfq_Pae_; pBADHfq_PaeS65C_-Flag). The cells were grown at 37°C in LB medium. At an OD_600_ of 0.7, the synthesis of Hfq* was induced by addition of IPTG and arabinose to a final concentration of 1 mM and 0.02%, respectively. The cells were harvested after 2 h, the cell pellet was resuspended in lysis buffer (50 mM Tris–HCl pH 8.0, 1.5 M NaCl, 250 mM MgCl_2_, 1 mM PMSF, 1 mM β-mercaptoethanol, 1 mM EDTA, 10 μg/ml DNAse I, 10 μg/ml RNaseA), and the cells were lysed by sonication. First, Hfq* was highly enriched by heating and ammonium sufate precipitation followed by three consecutive chromatography steps as described in detail by Beich-Frandsen *et al.* (33). To further determine the ratio between wild type (wt) Hfq and Hfq_S65C_-Flag in the Hfq* mixture, Hfq* was captured with anti-Flag M2 magnetic beads (Sigma Aldrich) as specified by the manufacturer. Then, given the difference in size between Hfq wt and Hfq_S65C_-Flag, a western-blot analysis was performed with anti-Hfq antibodies to determine the ratio of Hfq wt and Hfq_S65C_-Flag. Both, the column purified Hfq* and Hfq*, captured with the anti-Flag M2 magnetic beads, showed a 1:1 ratio of Hfq wt and Hfq_S65C_-Flag (Supplementary Figure S1A). For the Cryo-EM studies, *Pae* Hfq was purified from *E. coli* strain JW4130F′Δ*hfq* harboring plasmid pKEHfq_Pae_ as described before (33).

Crc was purified from strain PAO1(pMMB67HE-6His-3C-Crc) by Ni-affinity chromatography followed by removal of the His_6_-tag with GST-HRV14-3C ‘‘PreScission’’ protease as described by Milojevic *et al.* (29).

### Single-molecule experiments

**Figure 2.**
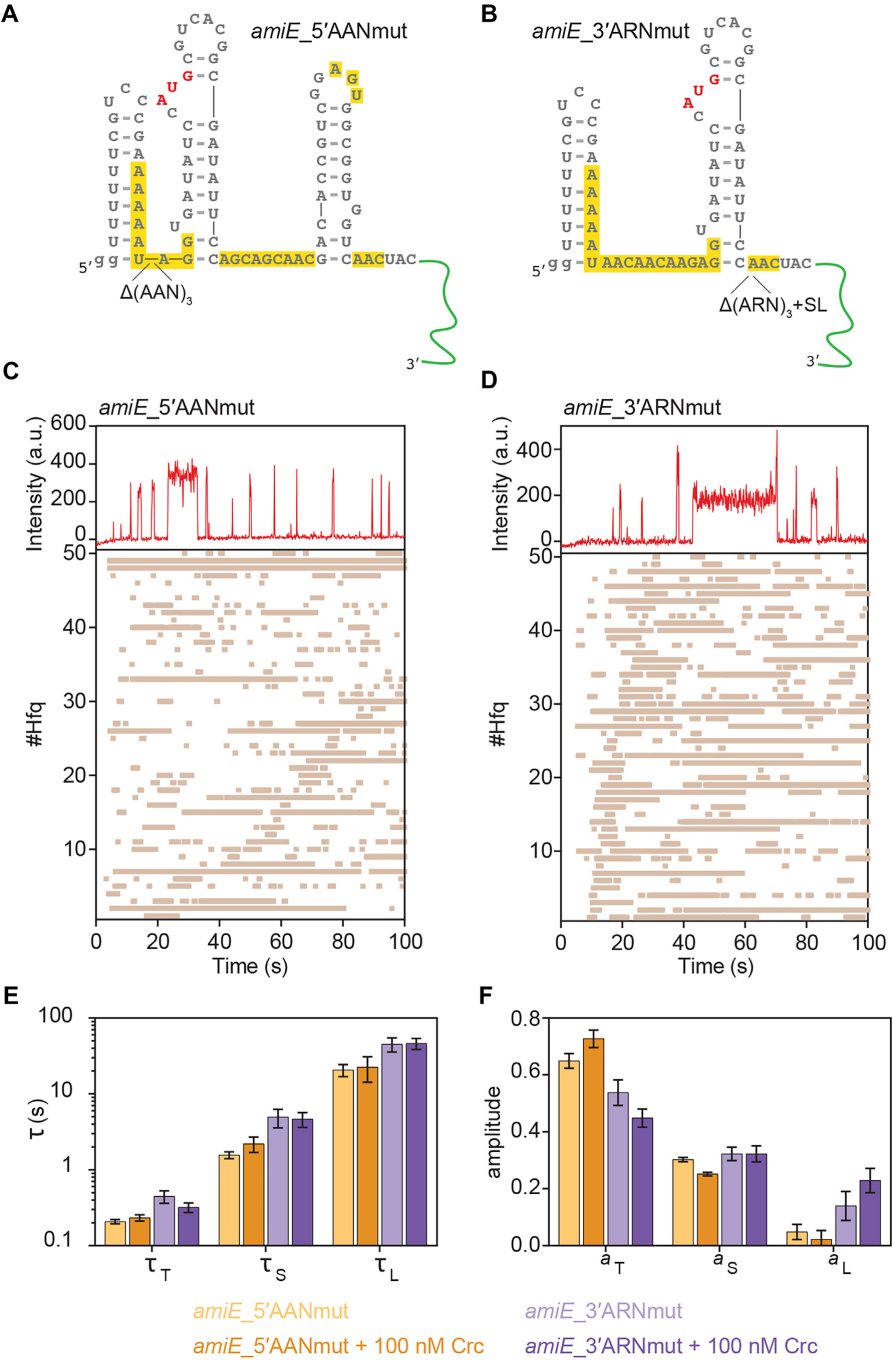
Hfq binds to the upstream and downstream (AA(R)N motifs. (A, B) Predicted secondary structure of the *amiE*_5′AANmut (**A**) and *amiE*_3′ARNmut (**B**) variants. The remaining AAN or ARN motifs in both RNAs are highlighted in yellow. The sequence of the extension hybridizing with Cy5-labeled DNA is indicated in green. SL, stem-loop. (C, D) Representative binding events between single Hfq* hexamers and *amiE*_5′AANmut (**C**) and *amiE*_3′ARNmut (**D**), respectively. Top panels, fluorescence intensity upon Cy5 excitation with 0.1 s frames. Bottom panels, rastergrams depicting the Hfq*/RNA interactions. 50 randomly selected traces are shown. Each horizontal bar represents an individual binding event. (**E, F**) Comparison of the lifetimes (τ_T (transient)_, τ_S (short)_ and τ_L (long)_; E) and the corresponding amplitudes (a_T (transient)_, a_S (short)_ and a_L (long)_; F) from maximum likelihood analysis of the dwell times of *amiE*_5′AANmut (orange) and *amiE*_5′AANmut (purple), respectively, on Hfq in the absence or presence of Crc. The error bars represent the s.d. of the mean determined from bootstrap analysis. Details of the number of observations and fit parameters can be found in Supplementary Figure S3 and Supplementary Table S1.

The RNA molecules used for the single-molecule experiments were prepared by *in vitro* transcription using T7 RNA polymerase, and then purified on 8 M urea 8% polyacrylamide gels. The DNA templates for transcription were obtained by extension of chemically synthesized overlapping oligodeoxyribonucleotides (Thermo Fisher Scientific) with Q5 DNA polymerase (NEB). The template for the *amiE*102 sub-sequence, which comprises nt –40 to +60 of *amiE* mRNA relative to the A (+1) of the AUG start codon, plus two additional Gs at the 5′end (Figure 1A) was generated by hybridization and extension of the *amiE*_F (5′-TAATACGACTCACTATAGGTTTTTTCGTCCCGAAAAAATAACAACAAGAGGTGATATCCATGCGTCACGGCGATATTTCCAGCAGC-3′) and *amiE_*R (5′CCTGTGTCCTGTGTGTCCTGTCCAAAGTGTGTCGTCCTGTAGTTGACCACCGCCACTCCGACGGTGTCGTTGCTGCTGGAAATATCGCCG-3′) oligonucleotides, while the *amiE*_5′AANmut RNA (Figure 2A) was obtained by using the *amiE*_AANmut_F oligonucleotide (5′- TAATACGACTCACTATAGGTTTTTTCGTCCCGAAAAAATAGGTGATATCCATGC-GTCACGGCGATATTTCCAGCAGC-3′) in combination with the oligonucleotide *amiE*_R. The *amiE*_3′ARNmut RNA (Figure 2B) was obtained by the combination of primers: *amiE*_61_F (5′-TAATACGACTCACTATAGGTTTTTTCGTCCCGAAAAAATAACAACAAGAGGTGATATCCATGCGTCACG-3′) and *amiE*_61-linker-R (5′-CCTGTGTCCTGTGTGTCCTGTCCA-AAGTGTGTCGTCCTGTAGTTGGAAATATCGCCGTGACGCATGGATATCACC-3′). The resulting RNAs contained the 3′end extension -//-5′-AGGACGACACACTTTGGACAGGACACACAGGACACAGG-3′, which is complementary to the SA5-Cy5 DNA oligonucleotide (5′-CCTGTGTCCTGTGTGTCCTGTCCAAAGTGTGTCGTCC-3′/3Cy5/; 34).

Single-molecule data were collected using a custom-built prism-based total internal reflection fluorescence (TIRF) microscope with an EMCCD camera (Andor). A 640 nm laser was used for Cy5 excitation. A selected field of view was recorded upon flow of Cy5-RNA for 3 minutes. The Cy5 emission signals were collected through a 60 × water immersion objective using custom software developed in the Ha Lab (smCamera).

The *amiE*102 RNA (250 nM) or variants thereof were added to the same concentration of the SA5-Cy5 DNA nucleotide and annealed by denaturation at 75°C for 5 min in 1× TNK buffer (10 mM Tris–HCl pH 7.5, 50 mM NaCl, 50 mM KCl), refolded at 37°C for 15 min, and equilibrated at 20°C. DDS-coated quartz slides were pre-treated with biotinylated BSA (0.2 mg/ml), Tween-20 (0.2%) and Neutravidin (0.1 mg/ml) as previously described (35). Biotinylated anti-FLAG antibodies (1mg/ml) were 12.500× diluted in imaging buffer (10 mM Tris–HCl pH 7.5, 50 mM NaCl, 50 mM KCl, 4 mM Trolox, 0.01% octaethylene glycol monododecyl- ether (Nikkol), 0.8% glucose, 2 U RNasin Plus) and applied to the slide for 5 min. Unbound antibody was removed by washing with imaging buffer. The immobilized antibody was incubated with 20 nM Hfq* for 5 min, then washed with imaging buffer supplemented with 165 U/ml glucose oxidase. We used unlabeled protein because we noticed that labeling of Pae Hfq* changes the kinetics of RNA binding. The RNA/DNA complexes were diluted to 1 nM in imaging buffer containing glucose oxidase and added to the slide channel in the absence or presence of 100 or 500 nM Crc. As judged from native gel mobility shift assays, the Cy5 DNA oligomer was not displaced from the RNA by Hfq (not shown). The time trajectories of single molecules were extracted from the CCD images using custom IDL code developed in the Ha lab. The Cy5 molecules were selected based on spots appearing in the acceptor channel during the whole duration of the movie.

### Single-molecule data analysis

The dwell times were collected using custom Matlab code. To estimate the lifetimes and associated errors, un-binned distributions were fit using maximum likelihood methods (36) to equations containing two or three exponential terms (Equations 1 and 2, respectively). In both equations, *t_m_* is the minimum resolvable time interval in the experiment, *t_x_* represents the duration of the experiment, τ_1_, τ_2_, τ_3_ represent characteristic lifetimes and *a, a_1_*, and *a_2_* are the fitted amplitudes; *a*_3_ = 1– *a*_1_– *a*_2_. The amplitudes of each term of an exponential distribution represent the proportion of detected events that occur with a given rate constant (1/τ). The amplitude values are between 0 and 1.

(1) $$\begin{eqnarray*}&&\frac{1}{{a \times \left( {{e^{ - \frac{{{t_m}}}{{{{\rm{\tau }}_1}}}}} - {e^{ - \frac{{{t_x}}}{{{{\rm{\tau }}_1}}}}}} \right) + \left( {1 - a} \right) \times \left( {{e^{ - \frac{{{t_m}}}{{{{\rm{\tau }}_2}}}}} - {e^{ - \frac{{{t_x}}}{{{{\rm{\tau }}_2}}}}}} \right)}} \nonumber\\ &&\quad\times \left( {\frac{a}{{{{\rm{\tau }}_1}}} \times {e^{ - \frac{t}{{{{\rm{\tau }}_1}}}}} + \frac{{1 - a}}{{{{\rm{\tau }}_2}}} \times {e^{ - \frac{t}{{{{\rm{\tau }}_2}}}}}} \right)\end{eqnarray*}$$

(2) $$\begin{eqnarray*}&&\frac{1}{{{a_1} \times \left( {{e^{ - \frac{{{t_m}}}{{{{\rm{\tau }}_1}}}}} - {e^{ - \frac{{{t_x}}}{{{{\rm{\tau }}_1}}}}}} \right) + {a_2} \times \left( {{e^{ - \frac{{{t_m}}}{{{{\rm{\tau }}_2}}}}} - {e^{ - \frac{{{t_x}}}{{{{\rm{\tau }}_2}}}}}} \right) + \left( {1 - {a_1} - {a_2}} \right) \times \left( {{e^{ - \frac{{{t_m}}}{{{{\rm{\tau }}_3}}}}} - {e^{ - \frac{{{t_x}}}{{{{\rm{\tau }}_3}}}}}} \right)}}\nonumber\\ &&\quad \times \left( {\frac{{{a_1}}}{{{{\rm{\tau }}_1}}} \times {e^{ - \frac{t}{{{{\rm{\tau }}_1}}}}} + \frac{{{a_2}}}{{{{\rm{\tau }}_2}}} \times {e^{ - \frac{t}{{{\tau _2}}}}} + \frac{{1 - {a_1} - {a_2}}}{{{{\rm{\tau }}_3}}} \times {e^{ - \frac{t}{{{{\rm{\tau }}_3}}}}}} \right)\end{eqnarray*}$$

Errors in fitted parameters were determined by bootstrapping 1000 random samples of initial data, fitting the resultant values with a normal distribution, and determining the standard deviation, σ. The error for *a_3_* was obtained by error propagation (Equation 3).

(3) $$\begin{equation*}{\rm{\;}}{\sigma _{{a_3}}} = \sqrt {{{({\sigma _{{a_1}}})}^2} + {{\left( {{\sigma _{{a_2}}}} \right)}^2}} \;\end{equation*}$$

Error bars in the histograms visualizing the maximum likelihood fits represent the standard deviation (Equation 4), where *N* is the number of events and *P* is the event probability.

(4) $$\begin{equation*}\sigma \; = \sqrt {NP\left( {1 - P} \right)} \;\end{equation*}$$

To assess the significance of the observed changes in the absence *versus* the presence of Crc, a two-tailed, unpaired *t*-test was performed to calculate the *P* value between given conditions using GraphPad Prism 6.

### Cryo-EM sample preparation, imaging and data analysis

**Figure 3.**
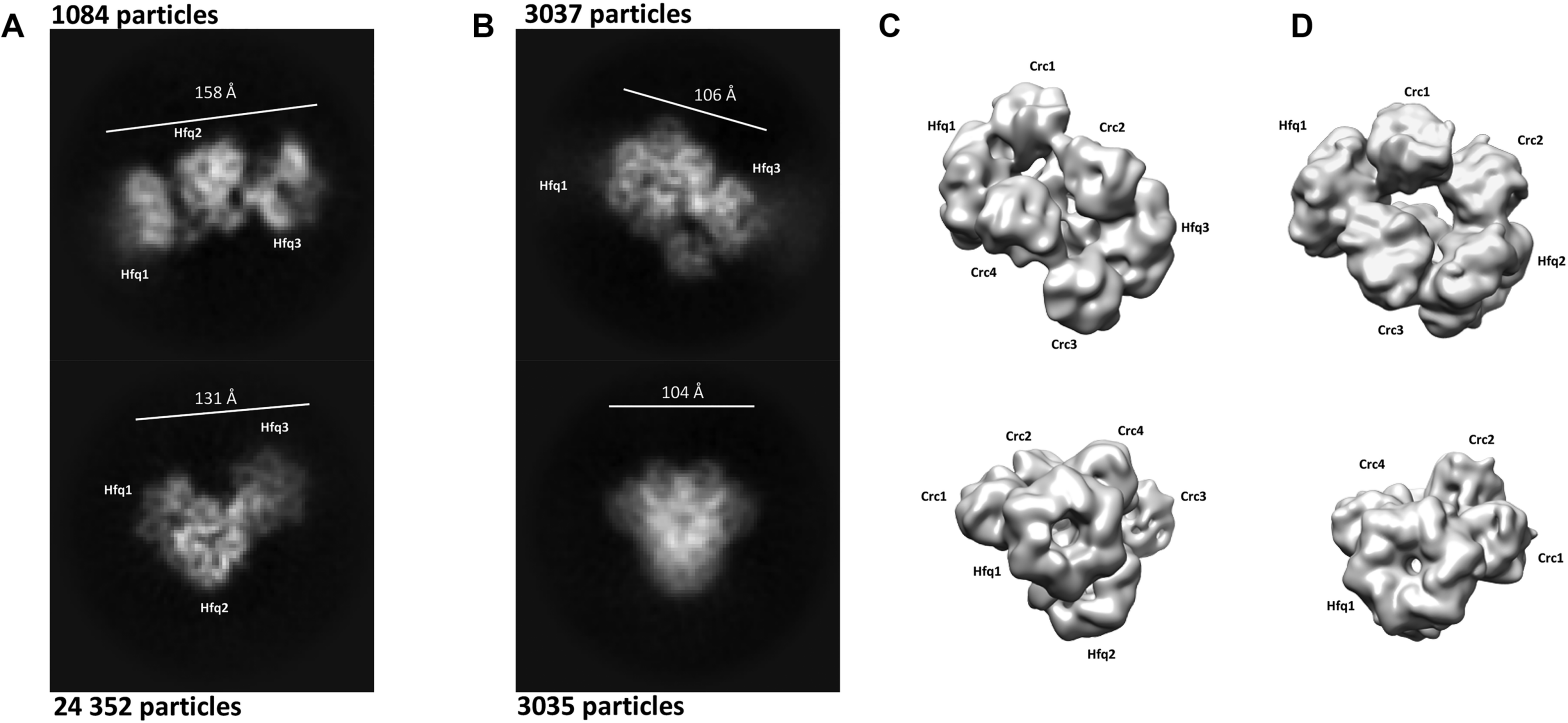
2D class averages of Hfq/Crc/*amiE*105 complexes at a ratio of 2:1:1 (**A**, Crc deficit) and 2:11:1 (**B**, Crc excess). The top and bottom 2D class averages shown in panel A correspond to two stoichiometrically identical assemblies with different relative orientations of the Hfq-components, behaving like ‘loose beads on a string’. The extendedness is 158 and 131 Å for the top and bottom class averages, respectively. The top and bottom 2D class averages in panel B correspond to different orientations of the same, well-defined and compact Hfq/Crc/*ami*E105 species in the sample. The extendedness is 106 and 104 Å for the top and bottom class averages, respectively. Particle numbers contributing to each class average are annotated. (**C**) 3D reconstructions of the complex shown in panel B, lowpass filtered to 15 Å resolution, and oriented according to the corresponding 2D class averages in panel B. Four Crc molecules (Crc 1–4) form bridging densities between the three Hfq hexamers in the assembly (Hfq 1–3). (**D**) For comparison, 3D reconstructions of the highest order Hfq/Crc/*amiE_6ARN_* assemblies as shown by Pei *et al.* (24), lowpass filtered to the same resolution and depicted at the same sigma value as the maps in panel C.

For the Cryo-EM studies, *amiE*105 RNA (nt –45 to +60 of *amiE* mRNA with respect to the A (+1) of the AUG start codon was prepared by *in vitro* transcription using T7 RNA polymerase, and then purified on a 8M urea 8% polyacrylamide gel. The DNA template was amplified by PCR using the primer pair R185 (5′-TCTAGACGTAATACGACTCACTATAGGGCCTT-TTTTCGTCCCGAAAAAATAACAAC-3′) and Z172 (5′-GTAGTTGACCACCGCCACTC-3′), wherein the forward primer R185 contained the T7 promoter sequence. When compared with *amiE*102, the yield obtained with the *amiE*105 template was ∼2-fold higher. The RNA *amiE*105 is identical to *amiE*102 except for the 3′extension and that the artificial two G-nucleotides at the 5′-end of *amiE*102 RNA are replaced by 5′-GGGCC-3′.., which is identical to the authentic sub-sequence of *amiE*. For Hfq and Hfq/Crc assembly on *amiE*105, the RNA was annealed at 50°C for 3 minutes. Hfq and Hfq/Crc were mixed at 2:1 and 2:11 ratios prior to addition of *amiE*105 (800 nM final concentration). After incubation on ice for 1 h, the mixture was diluted 7-fold before loading onto grids. Graphene oxide (GO) grids were prepared from regular Quantifoil r1.2/1.3 grids. A 2 mg/ml graphene oxide dispersion (Sigma Aldrich) was diluted 10-fold and spun at 300 × *g* for 30 s to remove larger aggregates. The dispersion was then diluted 10-fold before applying 1 μl to glow-discharged grids (0.29 mbar, 15 mA, 2 min, Pelco Easiglow glow discharger). After drying, 3 μl of the sample was applied to the GO grids and after 30 s of incubation, excess sample was blotted away and frozen in liquid ethane (Vitrobot markIV (FEI)). The grids were screened on a 200 kV Talos Arctica (FEI) (Cryo-EM facility, Department of Biochemistry, University of Cambridge), and datasets collected on a 300 kV Titan Krios (FEI) with a K3 (Gatan) direct electron detector (BioCem facility, Department of Biochemistry, University of Cambridge). For the 2 Hfq/1 Crc/1 *amiE*105 dataset, a set of 1007921 particles was extracted by Warp (37), and further processed with CryoSparc v2.15 (38) *via* multiple rounds of 2D classification. Due to significant preferential orientation bias of the particles in vitreous ice, as illustrated by the large discrepancy in particle numbers contributing to the 2D class averages in Figure 3A, no 3D reconstructions were generated. For the 2 Hfq/11 Crc/1 *amiE*105 dataset, 2757685 particles were extracted by Warp (37), and further processed with CryoSparc v2.15 (38) *via* multiple rounds of 2D classification and 3D heterogeneous refinement. Prior to heterogeneous refinement, a 3D *ab initio* reconstruction was generated in CryoSparc v2.15 from a subset of the particles. The maps from heterogeneous refinement were lowpass filtered to 15 Å for depiction, and a selection of 2D class averages was used to measure compaction of the Hfq/*amiE*105 intermediate upon Crc binding (Figure 3B).

### *In vitro* translation

**Figure 4.**
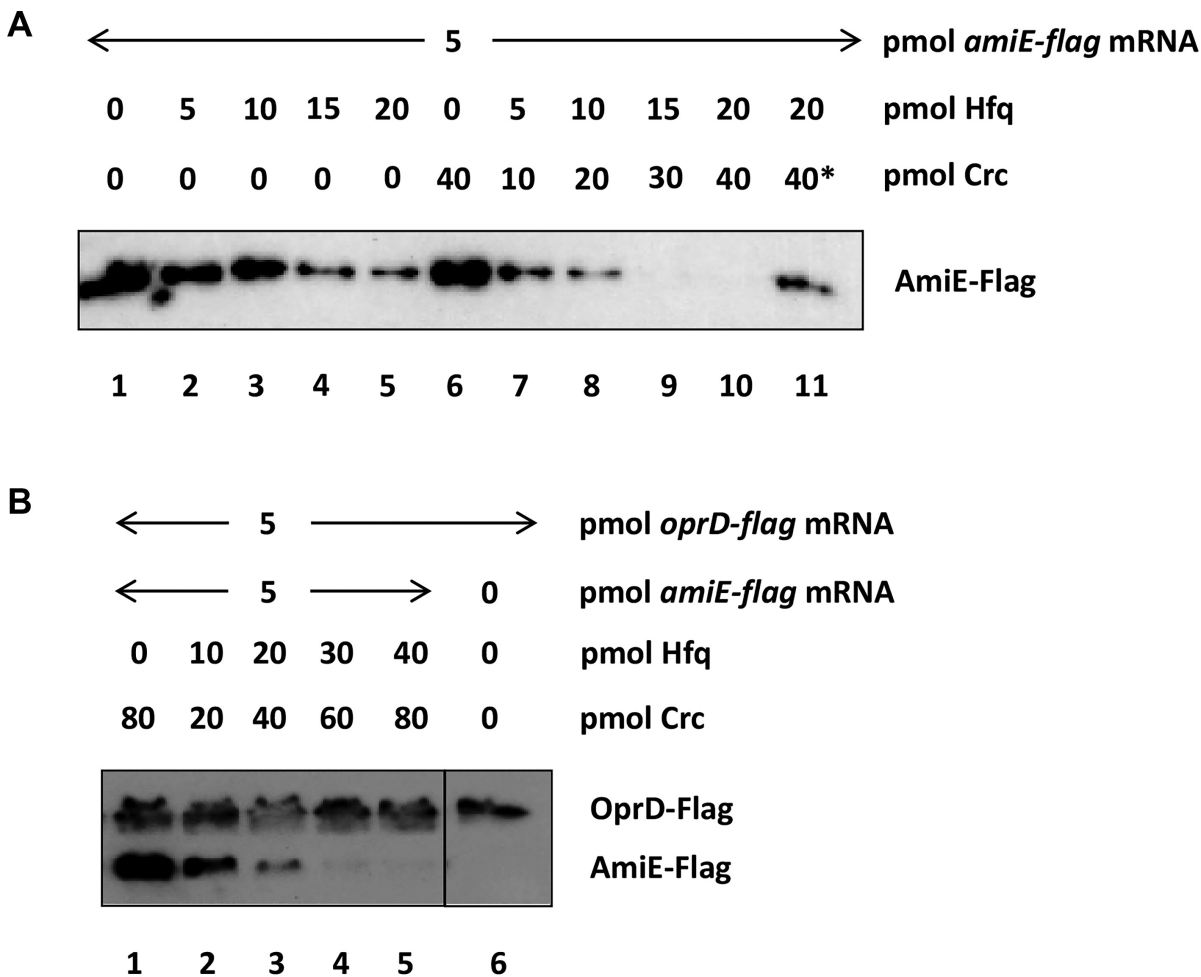
Enhancement of translational repression of *amiE-flag* mRNA by Crc *in vitro* (**A**) Repression of *amiE-flag* translation by Hfq and Hfq/Crc. Lane 1, the translational yield of AmiE-Flag protein in the absence of Hfq was set at 100%. Lanes 2–5, inhibition of *amiE-flag* mRNA translation by 25 ± 2.7%, 33 ± 0.2%, 80 ± 7.2% and 89 ± 0.4% in the presence of 5, 10, 15 and 20 pmol Hfq, respectively. Lane 6, the translational yield of AmiE-Flag protein in the absence of Hfq and in the presence of 8-fold molar excess of Crc over mRNA was set at 100%. Lanes 7–10, inhibition of *amiE-flag* mRNA translation by 85 ± 0.9%, 93 ± 4.1%, 100 ± 0.1% and 100 ± 0.3% in the presence of 5, 10, 15 and 20 pmol Hfq, respectively. Crc was added in all assays (lanes 7–10) in two-fold molar excess over Hfq. Lane 11, Crc was denatured for 15 min at 90 °C prior to addition to the *in vitro* translation assay (Indicated by 40*). The experiment was performed in duplicate. The result from one representative experiment is shown. (**B**) *In vitro* translation competition assay programmed with *amiE-flag* and *oprD*-*flag* mRNA in the presence of Hfq/Crc. Lane 1, translation of *amiE*_Flag_ and *oprD*_Flag_ mRNA in the presence of an 8-fold molar excess of Crc over the mRNAs. Lanes 2–5, translation of *amiE-flag* and *oprD*-*flag* in the presence of increasing concentrations of Hfq/ Crc, the latter of which was added in all assays in 2-fold molar excess over Hfq hexamers. Lane 6, *in vitro* translation of *oprD*-*flag* mRNA. The assays were performed in duplicate. The result from one representative experiment is shown.

*In vitro* translation was performed with the PURExpress *in vitro* protein synthesis kit (New England BioLabs) in the presence of 20 U RiboLock RNase Inhibitor (Thermo Fisher Scientific). 5 pmol of *in vitro* transcribed *amiE-flag* mRNA (2) was used in a 12.5 μl reaction and 5 pmol of either *in vitro* transcribed *amiE-flag* and *oprD-flag* mRNA were used in a 25 μl reaction. For *in vitro* transcription of *oprD-flag* mRNA, first, a PCR fragment was generated with the primer pair Y133 (5′-TCTAGACGTAATACGACTCACTATAGGCTAGCCGTCACTGCGGCAC-3′) and A131 (5′-TTTTTTCTATTATCAtcacttgtcgtcatcgtctttgtagtcCAGGATCGACAGCGG -3′). The forward primer contained a T7 promoter sequence and the reverse primer contained the Flag-tag encoding sequence (lower-case letters). The amplified PCR fragment was use as template in an *in vitro* transcription reaction using the AmpliScribe T7-Flash Transcription Kit (Epicentre Biotechnologies). Increasing amounts of purified Hfq protein as indicated in Figure 4 were added alone or in the presence of a 2-fold molar excess of Crc protein. The protein amount was doubled in the reactions were both mRNAs were present. The proteins and mRNAs were added simultaneously. After 1 h of incubation at 37°C, 5 μl of the reaction was mixed with 5 μl protein loading buffer and AmiE-Flag and OprD-Flag synthesis, respectively, was assessed by Western-blotting. The proteins were separated on a 12% SDS-polyacrylamide gel and blotted onto a nitrocellulose membrane. The blot was blocked with 5% dry milk in TBS buffer, and then probed with rabbit anti-Flag-tag polyclonal antibodies (Antibodies Online GmbH) and anti-rabbit IgG HRP-linked antibody (Cell Signaling Technology). The antibody-antigen complexes were visualized with the Clarity Max^TM^ Western ECL Substrate (BioRad). The quantification of the signals was performed with the Volume Tools of ImageLab software 5.2.1 (BioRad).

### Determination of *amiE* stability

**Figure 5.**
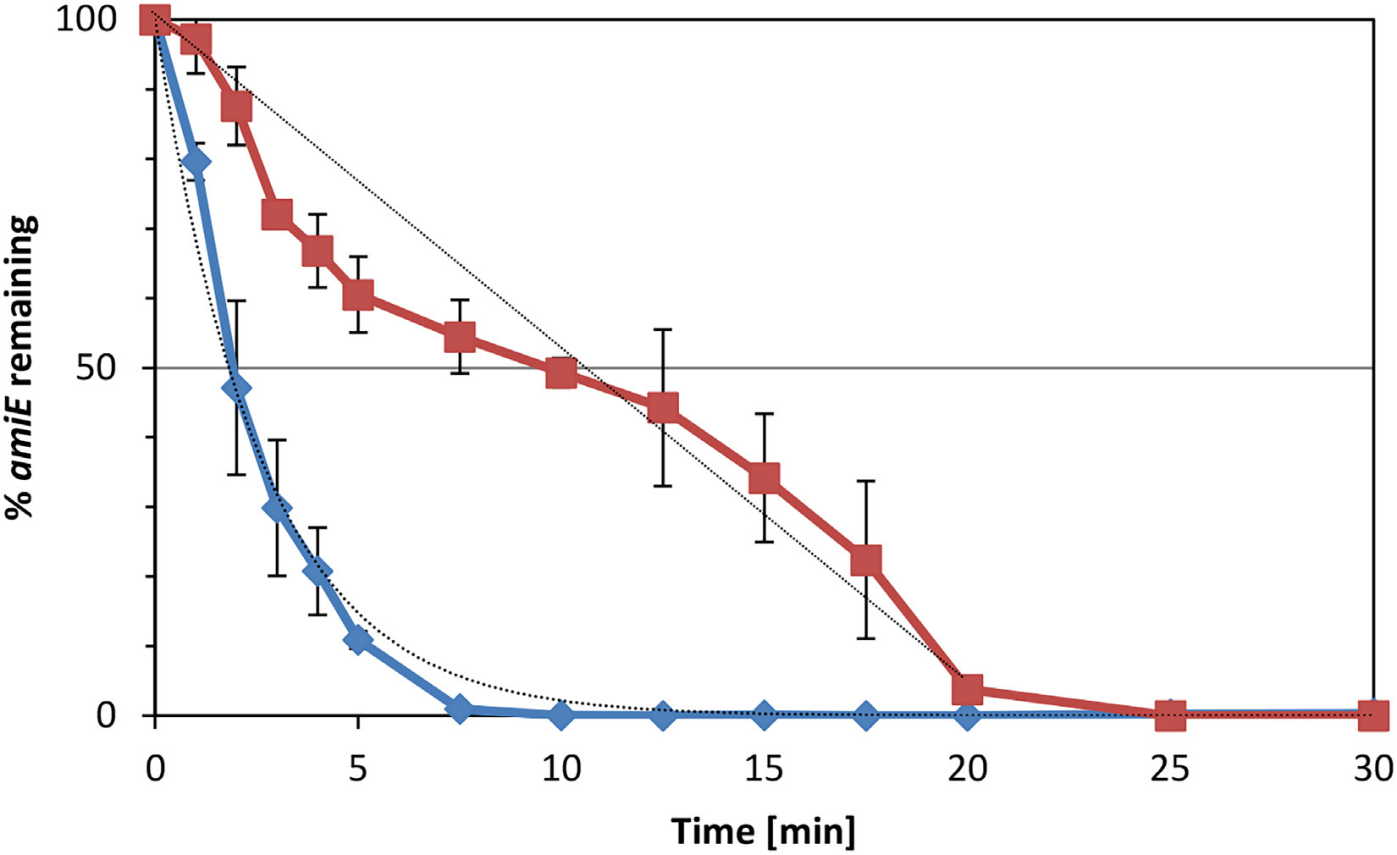
Acceleration of *amiE* mRNA decay by Crc *in vivo*. The strains PAO1 and PAO1Δ*crc* were grown in BSM medium supplemented with 40 mM acetamide and 40 mM succinate (+CCR). At an OD_600_ of 1.0, rifampicin was added to a final concentration of 100 μg/ml and samples were withdrawn for total RNA extraction at the times indicated. The remaining amounts of *amiE* mRNA in strains PAO1 (blue graph) and PAO1Δ*crc* (red graph) were normalized to that of 16S rRNA at different times after addition of rifampicin (see also Supplementary Figure S4). The average and standard-deviation from two biological replicates are presented.

The stability of *amiE* mRNA was determined in strains PAO1 (39) and PAO1Δ*crc* (25) grown in BSM medium supplemented with 40 mM acetamide and 40 mM succinate (+ CCR) to an OD_600_ of 1.0 followed by addition of rifampicin (100 μg/ml, final concentration). The samples were withdrawn at several times thereafter (Figure 5 and Supplementary Figure S4), and total RNA was extracted by using the hot phenol method (40). 2 μg of total RNA was used for cDNA synthesis of *amiE* and 16S rRNA. Finally, 2 μl of cDNA were used as a template for PCR amplification of *amiE* and 16S rRNA (internal control) as previously described (2). The quantification of the PCR signals was performed with the Volume Tools of ImageLab software 5.2.1 (BioRad). The values for the *amiE* signals were divided by the corresponding values of the 16S rRNA signals and time 0 was set as 100%. The degradation of *amiE* mRNA in PAO1 followed a first order kinetics and the half-life for was calculated by *t*_1/2_ = ln 2/*k*_decay_, whereas the *k*_decay_ values were extracted from the exponential trendlines (line of best fit) to the graphs determined in Excel. The *amiE* mRNA was not degraded with a first order kinetics in strain PAO1Δ*crc*. Therefore, the half-life was estimated by determining the slope (k*_decay_) from a linear trendline and the half-life was calculated by *t*_1/2_ = 50/k*_decay_.

## RESULTS

### Crc increases the population of stable Hfq/RNA complexes

Hfq/Crc assemblies are most likely forming on nascent mRNA (1), and thus on a longer sub-sequence than used in our previous studies (24). Therefore, we used a 100 nt *amiE* fragment to measure the protein binding dynamics by single molecule fluorescence co-localization. This RNA includes the authentic *amiE* mRNA sequence spanning nucleotides –40 to +60 with respect to the A (+1) of the AUG start codon, two extra 5′-terminal G nucleotides, and a 3′extension for annealing to a Cy5 labeled DNA oligonucleotide. (hereafter *amiE*102); Figure 1A and B). The *amiE*102 RNA comprises the proximal *amiE*_6ARN_ motif as well as further AA(R)N motifs for the distal site of Hfq that are located downstream of the AUG (2; Figure 1A).

To assess the impact of Crc on the Hfq/*amiE*102 complex a single-molecule fluorescence assay was established to monitor RNA binding to Hfq with 0.1 s resolution (Figure 1B). For these assays, we used mixed hexamers of Hfq wt and Hfq_S65C_-Flag (Hfq*) that contained both protomers in a 1:1 ratio (Supplementary Figure S1A). Neither the C-terminal Flag-tag nor the S65C substitution affected the interaction with Crc and *amiE*102 RNA, i.e. Hfq*/Crc/*amiE*102 complex formation (Supplementary Figure S1B) or the ability to repress a translational *amiE::lacZ* reporter gene (Supplementary Figure S1C). Hfq* was immobilized on a microscope slide *via* biotinylated anti-FLAG antibodies. The *amiE*102 mRNA fragment was transcribed with a 3′ extension that was hybridized to a Cy5-labeled DNA oligomer. Therefore, RNA binding to immobilized Hfq* could be observed by localization of the Cy5-labeled *amiE*102 RNA/DNA complex near the slide surface (Figure 1B).

The binding assays of *amiE*102 to immobilized Hfq* revealed multiple Cy5 signals appearing in the field of view (Figure 1C). They represent specific Hfq*/*amiE*102 interactions, as these events were not observed in control experiments lacking Hfq* or the anti-FLAG antibodies (Supplementary Figure S2A). Maximum likelihood fits of the dwell times for these interactions revealed that *amiE*102 forms two types of complexes with Hfq; unstable complexes with a lifetime of ∼2 s, and stable complexes with ∼60 s lifetime (Figure 1E and Supplementary Figure S2C). The unstable complexes were slightly more common than long-lived complexes during the first 3 min after RNA injection (*a*_short_ = 0.56 ± 0.05; Supplementary Table S1). The Cy5 photobleaching lifetime (173 s; Supplementary Figure S2B) was ∼3 times τ_L (long)_ (∼60 s), indicating that photobleaching might lead us to slightly underestimate the lifetimes of the most stable complexes, but does not account for the short-lived complexes that we observed.

Next, we tested whether Crc alters the binding of Hfq* to *amiE*102. The presence of 100 or 500 nM Crc did not change the lifetimes of the stable and unstable Hfq*/*amiE*102 interactions, suggesting that Crc does not affect the mode of Hfq/RNA binding (Figure 1D and E). However, Crc increased the population of stable complexes (*a*_long_ = 0.7 ± 0.04; Figure 1F). The higher concentration of Crc (500 nM) did neither affect the lifetimes nor the fractions of unstable and stable complexes (Figure 1E and F), suggesting that the complexes were saturated with Crc. Thus, in the presence of Crc, a higher fraction of the Hfq*/*amiE*102 complexes are long-lived, indicating that Crc locks Hfq onto *amiE*102 RNA, preventing the complexes from dissociating.

### Upstream and downstream AA(R)N motifs in *amiE* contribute to stable interactions with Hfq

Previous work revealed that the *amiE*_6ARN_ motif present in the translation initiation region of *amiE* (Figure 1A) is required for translational repression (2,25) and that it interacts with the distal face of Hfq (2,22). In addition, recent structural studies suggested that Hfq binding to the *amiE*_6ARN_ motif presents the RNA to Crc, thereby permitting the assembly of higher order Hfq/Crc/RNA complexes (24). To further assess the role of the *amiE*_6ARN_ motif for Hfq*/Crc/*amiE*102 assembly, three of the AAN triplets in the *amiE*_6ARN_ motif were deleted (hereafter, *amiE_*5′AANmut; Figure 2A). This deletion is not expected to impose significant changes in the predicted RNA secondary structure. The dwell time analyses revealed that the vast majority of the Hfq/*amiE_*5′AANmut binding events were transient and likely non-specific (∼0.2 s, Figure 2C, E, F). We also observed some short and long-lived complexes with lifetimes similar to those of *amiE*102 RNA (Figure 2E and Supplementary Figure S3). However, these short and long-lived complexes were much less prevalent for *amiE_*5′AANmut RNA than for *amiE*102 RNA (Figure 2F, Supplementary Table S1).

The *amiE*102 RNA also comprises Hfq binding motifs situated downstream of the start codon (Figure 1A). We therefore asked whether these motifs also contribute to the interactions with Hfq. To address this, a variant of *amiE*102 was generated that lacked the (ARN)_3_ motif and the distal hairpin comprising one putative ARN motif in the loop (hereafter, *amiE*_3′ARNmut; Figure 2B). The *amiE*_3′ARNmut RNA also formed both short- and long-lived complexes (Figure 2D and E). However, like *amiE_*5′AANmut, *amiE*_3′ARNmut formed noticeably fewer stable complexes with Hfq than *amiE*102, albeit marginally more than *amiE_*5′AANmut (*a*_L_, 0.14 ± 0.05 versus 0.05 ± 0.03; Figure 2F). Thus, Hfq is more likely to stably interact with the upstream *amiE*_6ARN_ motif than with the downstream (ARN)_3_ motif. Nevertheless, most binding events between *amiE*_3′ARNmut and Hfq were transient and non-specific (∼0.4 s; Figure 2F), indicating that both A-rich motifs are recognized by Hfq and that they both contribute to its stable association with *amiE*102.

When Crc was injected together with *amiE_*5′AANmut RNA, no change in binding was noted (Figure 2E and F; Supplementary Table S1). Crc only slightly decreased the fraction of non-specific *amiE*_3′ARNmut RNA complexes in favor of stable Hfq complexes (a_L_, 0.23 ± 0.04 *versus* 0.14 ± 0.05; Supplementary Figure S3A). These results indicated that Crc can only stabilize Hfq/RNA complexes when Hfq suitably interacts with the AA(R)N motifs located up- and downstream of the *amiE* start codon, indicating that Crc repression depends on sequence-specific recognition of the mRNA by Hfq.

### The presence of Crc results in compact Hfq/Crc/*amiE* assemblies

Next, Cryo-EM studies were performed to visualize the impact of Crc on Hfq/*amiE*105 complexes. 2D classification of Hfq/*amiE*105 assemblies indicated the presence of up to three Hfq hexamers that interacted with *amiE*105 RNA. The extended assemblies appeared as ‘loose beads on a string’ and revealed different relative orientations of Hfq in the folding intermediate (Figure 3A). The preferred particle orientations on the grid did not allow for interpretable 3D maps to be reconstructed, but the extendedness of the sub-assemblies could be measured directly from the 2D class averages with 158 Å and 131 Å for the top and bottom classes in Figure 3A, respectively.

When Crc was added to Hfq and *amiE*105, its presence resulted in more compact Hfq/Crc/*amiE*105 assemblies, consisting of complexes with apparent compositional stoichiometries of 3 Hfq: 4 Crc: 1 *amiE*105 (Figure 3B; Dendooven *et al.*, unpublished). This increased compactness of the Hfq/Crc/*amiE*105 assemblies was apparent from a decreased extendedness of the 2D class averages with 106 and 104 Å for the top and bottom class averages in Figure 3B, respectively. The 2D class average in the top panel of Figure 3B displays the greatest intramolecular length in the Hfq/Crc/*amiE*105 assembly, which is significantly lower than the sizes measured from the 2D class averages of the Hfq/*amiE*105 assembly (Figure 3A). Lastly, the compactness is also rationalized by preliminary 3D reconstructions (Figure 3C), where Crc molecules form proteinogenic bridges between the Hfq hexamers in the complex, as was recently observed for the Hfq/Crc/*amiE_6ARN_* assembly (24; Figure 3D).

### Increased translational repression in the presence of Crc

Previous *in vivo* studies with the *Pae* strains PAO1 wt and PAO1Δ*crc* revealed that translational repression of different Hfq/Crc regulated reporter gene fusions was reduced ∼ 50% in the absence of Crc, qualifying Crc as a translational co-repressor (2,9). To assess more directly the contribution of Crc in Hfq-mediated translational repression, full length *amiE* transcript abutted to a Flag-encoding sequence was translated *in vitro* in the presence of increasing molar ratios of Hfq and Hfq/Crc, respectively. Although, a 4-fold molar excess of Hfq-hexamer over *amiE-flag* mRNA did result in significant translational repression, translation was not completely abolished (Figure 4A, lane 5). In contrast, a 3- and 4-fold molar excess of Hfq resulted in complete translational shut off in the presence of Crc (Figure 4A, lanes 9 and 10). To verify that Hfq/Crc repression is restricted to Crc targets such as *amiE*, an *in vitro* translation competition assay programmed with *amiE-flag* mRNA and *oprD-flag* mRNA was performed. The *oprD* translation was recently shown to be down-regulated by two Hfq-dependent small RNAs (10). Hfq alone or the presence of Hfq/Crc did not impact translation of *oprD* (9). Consistent with these observations, translation of *oprD-flag* mRNA was hardly affected in the presence of increasing concentrations of Hfq and Crc, whereas translation *of amiE-flag* mRNA ceased in the presence of Hfq/Crc (Figure 4B). These results suggested that co-binding of Hfq and Crc and the presumed assembly of higher order Hfq/Crc/RNA complexes, similar to those shown in Figure 3B and C, ensures efficient translational repression of the target mRNA.

### Accelerated decay of *amiE* mRNA in the presence of Crc

Translational repression of bacterial mRNAs very often results in their rapid degradation (41,42). Therefore, we next tested the stability of the *amiE* transcript in the presence and absence of Crc in strains PAO1 and PAO1Δ*crc*. Under conditions of CCR, the half-life of *amiE* mRNA was 1.7 ± 0.1 min in strain PAO1, which is in the presence of Crc. In contrast, the half-life increased >6-fold to 10.4 ± 0.9 min in strain PAO1Δ*crc* (Figure 5 and Supplementary Figure S4). Together with the results shown above, we conclude that Crc compacts and stabilizes Hfq/Crc/RNA assemblies, strengthening translational repression, which leads to rapid degradation of target mRNAs during CCR when their encoded functions are dispensable.

## DISCUSSION

In *Pae*, the concerted action of Hfq and Crc steers cell metabolism by governing the hierarchical utilization of carbon sources. Previous studies on the Hfq/Crc repressome strongly indicated that Crc works only in concert with Hfq, i.e. Hfq acts as the principle translational repressor and Crc has an ancillary role as a co-repressor (1,22,23). RNA-seq based comparative transcriptome analyses with the *Pae* mutant strains PAO1*hfq*- and PAO1*Δcrc* revealed many overlapping mRNA targets, regulation of which is governed by both, Hfq and Crc during CCR (22). In addition, a ChIP-seq approach showed that Hfq and Crc co-associate with nascent transcripts, whereas Crc was not observed on RNAs in the absence of Hfq (1). These *in vivo* findings are in accord with previous *in vitro* results, showing that Crc is devoid of RNA binding activity (29), and that the association of Crc with RNA depends on Hfq (21,22). Moreover, we have performed an RNA-seq based comparative transcriptome analysis with the *Pae* strains PA01Δ*hfq* and PA01Δ*hfq*Δ*crc* to verify that Crc does not exert an independent regulatory function. As shown in Supplementary Table S2, using standard thresholds of significance for the analysis only eight transcripts were differentially abundant in the presence of Crc in strain PA01Δ*hfq* when compared with strain PA01Δ*hfq*Δ*crc* under conditions of CCR. As none of these transcripts was identified by ChIP-seq using anti-Crc antibodies (1), it is rather unlikely that these genes are directly regulated by Crc. However, at this stage we cannot exclude that Crc interacts with another regulatory protein(s) that is involved in regulation of these transcripts.

**Figure 6.**
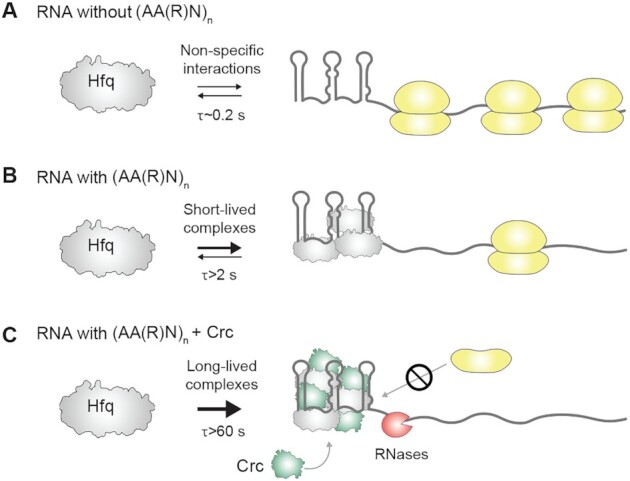
Model for stabilization of Hfq-mediated translation repression complexes by the co-repressor Crc. (**A**) Transient interactions of Hfq with RNAs lacking suitable (AA(R)N)_n_ motifs results in translation, as indicated by traversing 70S ribosomes (yellow). (**B**) The presence of canonical (AA(R)N)_n_ Hfq-binding motifs and the formation of short-lived Hfq/RNA complexes leads to moderate translational repression. (**C**) Binding of Hfq to (AA(R)N)_*n*_ motifs followed by a suitable presentation of the RNA to and recruitment of Crc results in stable repressive complexes that prevent formation of productive 30S translation initiation complexes and enhance opportunities to encounter the RNA degradation machinery. For further details, see text.

Although these studies underlined the cooperative action of Hfq and Crc, the question arises as to why two proteins are needed to efficiently repress translation of a target mRNA. To gain deeper insight into the mechanism of regulation, we developed a single-molecule assay that visualizes individual binding events on time scales of milliseconds. Because single-molecule studies enable characterization of transition states, they are a valuable tool for understanding molecular processes. In the context of Hfq, this technique was recently used to demonstrate unfolding of structured mRNAs during base-pairing with small RNAs (43). Here, the single-molecule assay revealed that the majority of Hfq/*amiE*102 complexes are intrinsically perturbed, which leads to a high dissociation rate (τ ∼2 s; Figure 6B). Our assays strongly corroborate the previous notion (24) that Crc engages transient, pre-organized Hfq/*amiE*102 complexes and shifts the equilibrium towards assemblies with increased stability (τ ∼ 60 s) so that the effector complex blocks translation more efficiently (Figure 6C). Thus, the cooperation of Hfq with Crc effectively stabilizes the repressive complex, excluding the 30S ribosome more effectively than can be achieved by Hfq alone (Figure 6C).

When compared with the Hfq/*amiE*105 complex (Figure 3A), the increased stability of the Hfq/Crc/*amiE*102 assemblies determined by the single molecule experiments can be readily reconciled with the 2D and 3D Cryo-EM averages of the complex formed with both abundant and sparse concentrations of Crc (Figure 3B and C). In the 3D reconstructions, 3 Hfq hexamers and 4 Crc protomers can be discerned that build a compact assembly (Figure 3C). In fact, the observed compaction is anticipated to increase buried surface areas of the complex and is a hallmark of increased thermodynamic stability. Moreover, the arrangement of the Hfq hexamers on *amiE*105 suggests that Hfq binding is not restricted to the *amiE*_6ARN_ motif upstream of the AUG but involves as well cognate sequences of the immediate coding region. This observation is supported by the single molecule experiments with *amiE*_3′ARNmut RNA that implicated the distal Hfq-binding motif(s) in formation of stable Hfq/*amiE*102 complexes (Figure 2).

How does Crc engage as a translational co-repressor? Recent Cryo-EM studies showed that there are limited contacts between Crc and the rim of Hfq (24). Crc mainly contacted exposed bases at the ‘N’ position of the ARN motif and the RNA backbone in the Hfq/Crc/*amiE*_6ARN_ complex (24). Consequently, we suggested that complex assembly occurs in a checklist-like manner through numerous small contacting surfaces, and when the RNA target is presented by Hfq in a specific, well-defined configuration. It can be assumed that the location of Hfq binding motifs as well as the length and structure of an RNA molecule determine the meandering of it around one or more Hfq hexamers. Thus, the presentation mode of the RNA substrate by Hfq might impact Crc assembly. Our studies with the *amiE_*5′AANmut and *amiE_*3′ARNmut RNAs support this notion. Hfq bound to these RNAs dynamically, forming mostly transient interactions (τ ∼ 0.2–0.4 s; Figure 6A). The lack of the three upstream AAN or the three downstream ARN motifs most likely prevented proper presentation of the RNA by Hfq, therefore the addition of Crc did not result in increased stability of the complex. Hence, this observation underlines the notion that Crc can only engage preformed Hfq/RNA complexes.

Autogenously acting translational repressors generally employ molecular mimicry and operate either by competing with initiating ribosomes or by an entrapment mechanism, whereby the formation of a productive translation initiation complex is impeded (44). A well-studied example of the first type is the *E. coli* threonyl-tRNA-synthetase (ThrRS), which binds as a dimer to the operator on its own mRNA so that the N-terminal domain sterically prevents ribosome binding (45). The second type of translational repressors are exemplified by *E.coli* ribosomal proteins, such as S4 and S15. The S4 autoregulatory feedback mechanism involves allosteric changes in the mRNA structure, affecting the distribution of translationally active and inactive mRNA (46). Similarly, S15 binding to a pseudoknot structure on its own mRNA prevents formation of an active 30S complex by abrogating productive interactions of the anti-codon of initiator tRNA and the start codon on mRNA (47). Regardless of the different mode of action, the affinity of S4 (48) and of ThrRS (49) for their own mRNA is similar as that for the 30S subunit for a typical ribosome binding site (48,50). Thus, low concentrations of the repressor may allow efficient feedback regulation.

In contrast, effective translational repression by Hfq/Crc of undesirable mRNAs, such as *amiE* during CCR, or translational regulation by RsmA (51) would benefit from a higher affinity of the repressor than that of the 30S subunit for target mRNAs. Indeed, the affinity of Hfq/Crc and RsmA for target mRNAs was ∼5-fold (22) and ∼2-fold higher (3), respectively, than that of 30S subunits for a typical ribosome binding site (48,50). The formation of higher order complexes as observed for Hfq/Crc (Figure 3) or dimers as inferred for *Pae* RsmA (52) and shown for *P. fluorescens* RsmE (18) appear to contribute to the stability of the complexes, and thus to an increased competition with 30S subunits. Moreover, these stable repressive complexes will most likely result in a translationally inactive mRNA that is kinetically trapped and subject to rapid degradation as shown for *amiE* mRNA (Figures 4, 5 and 6C).

Although Hfq is well-known to act with sRNAs, this study shows how Hfq can collaborate with another protein to down-regulate mRNA expression. Thus, to effect robust translational control Hfq appears to need a partner—either a sRNA or another protein—to lock it onto the mRNA. Finally, assembly of Hfq/Crc on nascent mRNAs may provide a means for efficient competition with small RNA substrates of Hfq. In the complexes shown in Figure 3B and C, it is most likely that sRNA binding sites on the proximal side and the rim of Hfq (53) are shielded by the winding mRNA. We hypothesize that several AA(R)N motifs in the 5′UTR and/or the immediate 5′coding region of a mRNA (Figure 1A) result first in assembly of several Hfq hexamers followed by suitable presentation of the mRNA to and assembly of Crc protomers. As previously suggested (22), the sequestration of sRNA binding sites on Hfq would allow the formation of stable repressive Hfq/Crc/mRNA complexes that are inaccessible to invading sRNAs.

## Supplementary Material

gkab510_Supplemental_File
