# Supplementary material for: Stabilization of Hfq-mediated translational repression by the co-repressor Crc in Pseudomonas aeruginosa
Source: Nucleic Acids Res. 2021 Jun 17;49(12):7075–87. doi: 10.1093/nar/gkab510 (PMC8266614; doi:10.1093/nar/gkab510)
Supplement: gkab510_Supplemental_File [file gkab510_supplemental_file.pdf]

Supplementary Materials and Methods

Stabilization of Hfq-mediated translational repression by the co-repressor Crc in *Pseudomonas aeruginosa*

Ewelina M. Malecka[2], Flavia Bassani[1*], Tom Dendooven[3*], Elisabeth Sonnleitner[1*], Marlena Rozner[1], Tanino G. Albanese[1], Armin Resch[1], Ben Luisi[3], Sarah Woodson[2§] and Udo Bläsi[1§]

[1]Department of Microbiology, Immunobiology and Genetics, Max Perutz Labs, University of Vienna, Vienna Biocenter (VBC), Dr. Bohrgasse 9/4, 1030 Vienna, Austria.

[2]Department of Biophysics, 3400 N. Charles Street, Johns Hopkins University, Baltimore, MD-21218, USA

[3]Department of Biochemistry, University of Cambridge, Cambridge CB2 1GA, UK

[§]Correspondence:

udo.blaesi@univie.ac.at

swoodson@jhu.edu

*Equal contribution

Keywords: Hfq, Crc, translational repression, carbon catabolite repression, *Pseudomonas aeruginosa*

TEXT S1 SUPPLEMENTARY INFORMATION

Supplementary Materials and Methods

RNA Sequencing

Total RNA was prepared from two biological replicates of strains PAO1Δ*hfq* and PAO1Δ*hfq*Δ*crc*, respectively, after growth in LB medium. Under these conditions, the levels of CrcZ RNA are low and CCR occurs (26). At an OD_{600} of 1.5, 10 ml samples were withdrawn and total RNA was extracted using the hot phenol method (40). Contaminating DNA was removed by Turbo^{TM} DNase (Thermo Scientific) treatment followed by phenol-chloroform (pH 5.5) extraction and ethanol precipitation. To remove ribosomal RNAs, the Microbe*Express*^{TM} Bacterial mRNA enrichment kit (Ambion) was used according to the manufacturer's instructions. Libraries were constructed using NEBNext® Ultra™ Directional RNA Library Prep Kit from Illumina. 100 base pair single end sequence reads were generated using Illumina HiSeqV4 SR100 at the Vienna BioCenter Core Facility (http://www.csf.ac.at). Sequencing adapter removal was performed with cutadapt (55). Trimmed reads were aligned on PAO1 genome (PAO1_NC_002516.2) using bowtie (56); a maximum of 2 mismatches were allowed among the first 28 nt of the reads. Multi-mappers are assigned to their best match and counted only once for the purpose of sample normalization Reads mapping on each gene of PAO1 (according to latest assembly: PAO1_GCF_000006765.1) were counted using HTSeq package for Python (57); all reads that were mapped to a gene (either fully or partially) were assigned to that gene. In contrast, reads that overlapped to more than one gene, were considerate ambiguous and discarded. Finally, R-package DESeq2 (58) was employed to normalize read counts among samples and calculate the relative difference between the gene expression in PAO1Δ*hfq*Δ*crc* in comparison to a single deletion of *hfq* as the reference strain. To minimize the effect of high variance in the quantification of low read-count genes, the resulting

logarithmic fold change (LFC) was reduced as described (57). All RNAs with a fold-change greater than 2 and a multiple testing adjusted p-value below 0.05 were considered to be differentially abundant. The raw sequencing data were deposited in the European nucleotide archive (ENA) as a study under the accession number PRJEB42408.

Construction of plasmids

Plasmid pKEHfq$_{Pae}$ was constructed for production of large quantities of Hfq$_{Pae}$. A 283-bp fragment of *hfq* (nt +1 to nt +283 with regard to the A (+1) of the start codon) fused to the RBS of phage T7 gene *10* was amplified by PCR using the oligonucleotides B119 (5' ttt*GAATTC*AATATAATAGTTTAACTTTAAGAAGGAGATATACATATG-3') and D119 (5'-ttt*CTGCAG*CCACCTGGGCGCTCAAAGAACAAAG-3') and plasmid pHfq$_{Pae}$ (2) as template. The resulting PCR product was cleaved with *Eco*RI and *Pst*I, and then ligated into the corresponding sites of plasmid pKES170 (59). The resulting plasmid pKEHfq$_{Paewt}$ harbors *hfq* under transcriptional control of P_{tac} and its translation is directed by the RBS of T7 gene *10*.

Plasmid pBADHfq$_{PaeS65C}$-Flag was generated to isolate the Hfq/Hfq$_{S65C}$-Flag heterooligomer (Hfq*) suitable for the single molecule experiments. First, the serine codon 65 was changed to a cysteine codon in the *hfq* gene encoded by plasmid pKEHfq$_{Pae}$. This was done by using the QuickChange site-directed mutagenesis protocol (Agilent Technologies). The oligonucleotides J163 (5'-CCGTGGTACCGTGCCGTCCG-3') and K163 (5'-CGGACGGCACGGTACCACGG-3') were first hybridized with pKEHfq$_{Pae}$ DNA and the entire plasmid was then amplified with Phusion DNA polymerase (Thermo Fisher Scientific). The parental plasmid templates were removed by *Dpn*I cleavage, and the mutated nicked circular

strands were transformed into *E. coli* strain XL1-Blue, generating pKEHfq$_{PaeS65C}$. This plasmid was used as a template for PCR amplification with the oligonucleotides S179 (5´-TTTTTGAGCTCAATATAATAGTTTAACTTTAAGAAGGAG--ATATACATATG-3´) and R179 (5'- AAAACTGCAGTCACTTGTCGTCATCGTCTTTGTAGTCAGCGTTGCCCG-GCTCG-3'). Oligonucleotide S179 contained a sequence derived from plasmid pET22b bearing the RBS of phage T7 gene *10* and a *Sac*I restriction site. Oligonucleotide R179 was designed to encode the Flag-tag sequence and a *Pst*I site. The resulting PCR fragment was cleaved with *Sac*I and *Pst*I and ligated into the corresponding sites of pBAD33 (60). In the resulting plasmid pBADHfq$_{PaeS65C}$-Flag transcription and translation of the *hfq*$_{\textit{Pae}\mathrm{S65C}}$-Flag gene is driven by the P$_{BAD}$ promoter and the RBS of T7 gene *10*, respectively.

Plasmid pMMB*hfq*$_{S65C\text{-}Flag}$ was generated by inserting the Hfq$_{S65C}$-Flag encoding sequence - flanked by *Sac*I and *Pst*I sites - of plasmid pBADHfq$_{PaeS65C}$-Flag into the cloning vector pMMB67HE (61).

Plasmid pMMB67HE-6His-3C-Crc was generated for production of large quantities of Crc in strain PAO1, the *crc* open reading frame fused to a N-terminal cleavable His$_6$-tag was amplified by PCR using the oligonucleotides W185 (5'-ttttAAGCTTAATAATTTTGTTTAACTTTAAGAAGGAGATATA-3') and X4 (5'-ATGCGGATCCTCAGATGCTCAACTGCCAG-3') and plasmid pETM14lic-His$_6$Crc (29) as template. The resulting PCR fragment was cleaved with *Hind*III and *Bam*HI and then ligated into the corresponding sites of plasmid pMMB67HE (61). The resulting plasmid pMMB67HE-6His-3C-Crc harbors *crc* under transcriptional control of P$_{tac}$.

Electrophoretic mobility shift assays (EMSA)

10 nM 5'-end labelled [^{32}P]-*amiE*102 RNA was incubated for 15 min at 37°C in ES buffer (10 mM Tris pH 8.0, 10 mM KCl, 40 mM NaCl and 1 mM $MgCl_2$) in the absence or presence of 80 nM Hfq*-protein and 0 or 320 nM Crc protein as specified in Supplementary Figure S1B in a total volume of 10 µl. Immediately before loading, the samples were mixed with 2 µl loading dye (25 % glycerol, 0.02 % bromphenol blue, 0.02 % Xylene cyanol), and then separated on a 4 % polyacrylamide gel using Tris-borate buffer. The radioactively labelled bands were visualized with a Typhoon FLA 9500 laser scanner (GE Healthcare). The proteins and the RNA were then semi-dry electro-blotted onto a double layer of membranes (nitrocellulose membrane and nylon membrane) in transfer buffer (25 mM Tris pH 8.3, 192 mM glycine, 20 % methanol). The transfer of the RNA was ensured by inspection of the membranes with a Typhoon FLA 9500. The nitrocellulose membrane was then blocked with 5 % dry milk in TBS buffer and probed with rabbit anit-Hfq (Pineda) and rabbit anti-Crc (Pineda) antibodies, respectively. The antibody-antigen complexes were visualized with anti-rabbit IgG HRP-linked antibodies (Cell Signaling Technology) and the Clarity Max™ Western ECL Substrate (BioRad).

Supplementary References

55. Martin, M. (2011) Cutadapt removes adapter sequences from high-throughput sequencing reads. *EMBnet journal*, **17**, 10-12.

56. Langmead, B., Trapnell, C., Pop, M., and Salzberg, S.L. (2009) Ultrafast and memory-efficient alignment of short DNA sequences to the human genome. *Genome Biol*, **10,** R25.

57. Anders, S. Pyl, P.T., and Huber, W. (2015) HTSeq—a Python framework to work with high-throughput sequencing data. *Bioinformatics*, **31**, 166–169.

58. Love, M.I., Huber, W., and Anders, S. (2014) Moderated estimation of fold change and dispersion for RNA-seq data with DESeq2. *Genome Biol*, **15,** 550.

59. Lüttmann, D., Heermann, R., Zimmer, B., Hillmann, A., Rampp, I.S., Jung, K., and Görke, B. (2009) Stimulation of the potassium sensor KdpD kinase activity by interaction with the phosphotransferase protein IIA(Ntr) in *Escherichia coli*. *Mol Microbiol*, **72**, 978-994.

60. Guzman, L.M., Belin, D., Carson, M.J., and Beckwith, J. (1995) Tight regulation, modulation, and high-level expression by vectors containing the arabinose PBAD promoter. *J Bacteriol*, **177**, 4121-4130.

61. Fürste, J.P., Pansegrau, W., Frank, R., Blöcker, H., Scholz, P., Bagdasarian, M., and Lanka, E. (1986) Molecular cloning of the plasmid RP4 primase region in a multi-host-range *tacP* expression vector. *Gene*, **48**, 119-131.

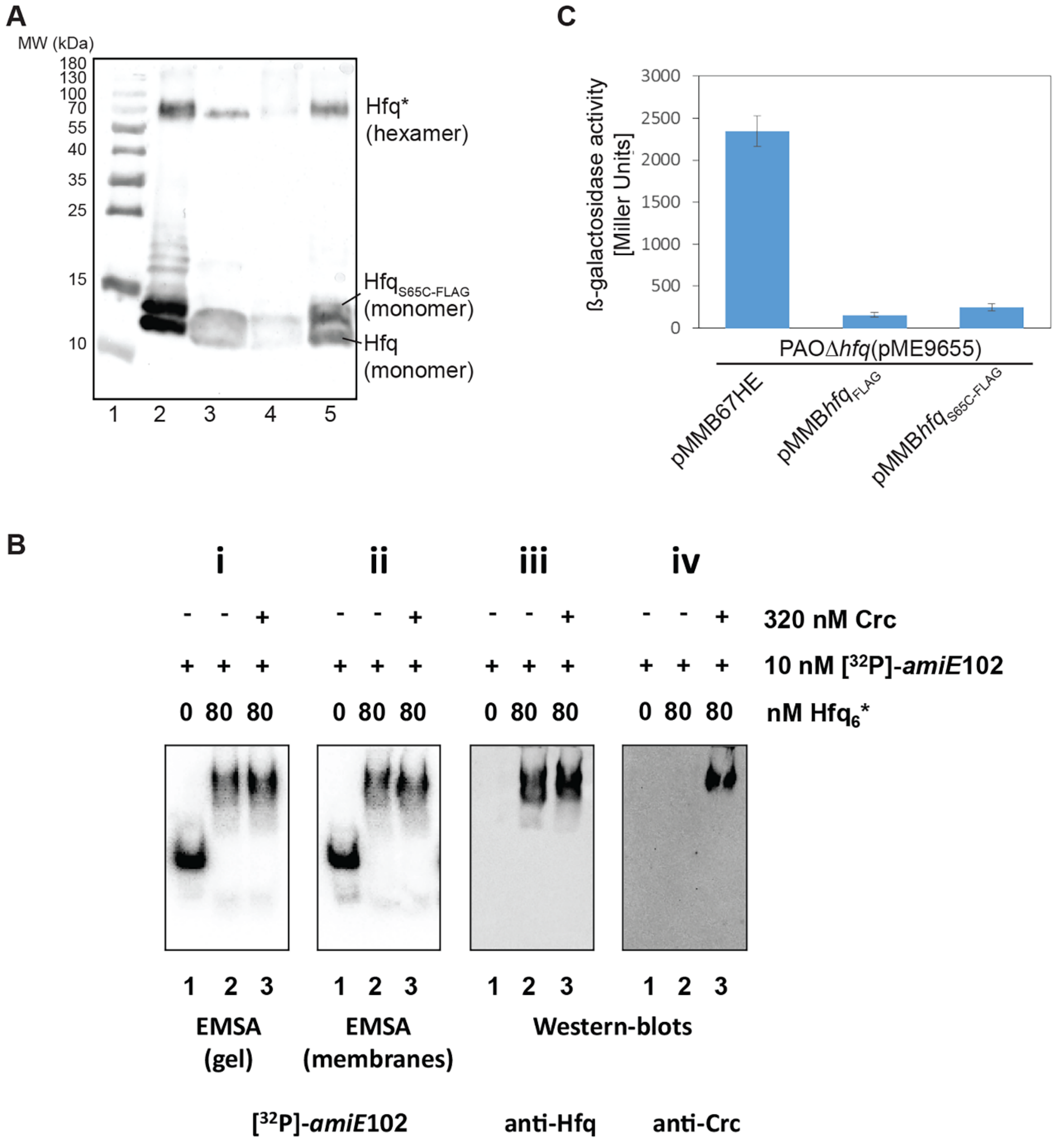

Supplementary Figure S1. (**A**) Capturing of Hfq* by anti-Flag magnetic beads. Hfq* was first enriched as described by Beich-Frandsen et al. (33). 200 pmol of purified Hfq* were incubated at 37 °C for 15 min, then 40 µl of anti-Flag M2 magnetic beads were added and the incubation was continued overnight at 4°C on a rotating wheel. The beads were washed three times, and bound Hfq* was eluted by incubation of the beads for 8 min at 95°C in 50 µl of SDS loading dye. 5µl of

each step were analyzed by Western-blotting using anti-Hfq specific antibodies (Pineda). Lane 1, molecular weight standards. Lane 2, input of purified Hfq*. Lane 3, unbound protein. Lane 4, wash fraction. Lane 5, Eluate fraction. The position of the Hfq* hexamer and of Hfq_{S65C}-Flag and Hfq (wt) monomers are marked. (**B**) Co-localization of Hfq*, Crc and *amiE*102 RNA visualized by an electrophoretic mobility shift assay (EMSA). (i) EMSA) with [^{32}P]-*amiE*102 RNA in the absence (lane 1) and presence of 80 nM Hfq* hexamer (lanes 2 and 3) and in the absence (lanes 1 and 2) and presence of 320 nM Crc protein (lane 3). (ii) Transfer of proteins and RNA onto a membrane bilayer of nitrocellulose and nylon. (iii and iv) Immunodetection of Hfq* (iii) and Crc (iv) with anti-Hfq (iii) and anti-Crc (iv) antibodies, respectively. (**C**) Repression of *amiE*::*lacZ* translation by Hfq-Flag and Hfq_{S65C}-Flag variants. The PAO1Δ*hfq* strains harboring plasmid pME9655 (2), encoding the *amiE*::lacZ translational reporter gene fusion and either plasmid pMMB67HE (parental vector; (2)), pMMBhfq_{Flag} (encodes Hfq-Flag; (2)) or plasmid pMMB$hfq_{\mathrm{S65C\text{-}Flag}}$ (encodes Hfq_{S65C}-Flag; Supplementary Materials) were grown to an OD_{600} of 1.5 in BSM medium supplemented with 40 mM succinate to establish CCR. Then, the cells were harvested and the ß-galactosidase activities were determined as described (2). The bars depict ß-galactosidase values conferred by the translational *amiE*::*lacZ* fusion encoded by plasmid pME9655. The error bars represent standard deviations from three independent experiments.

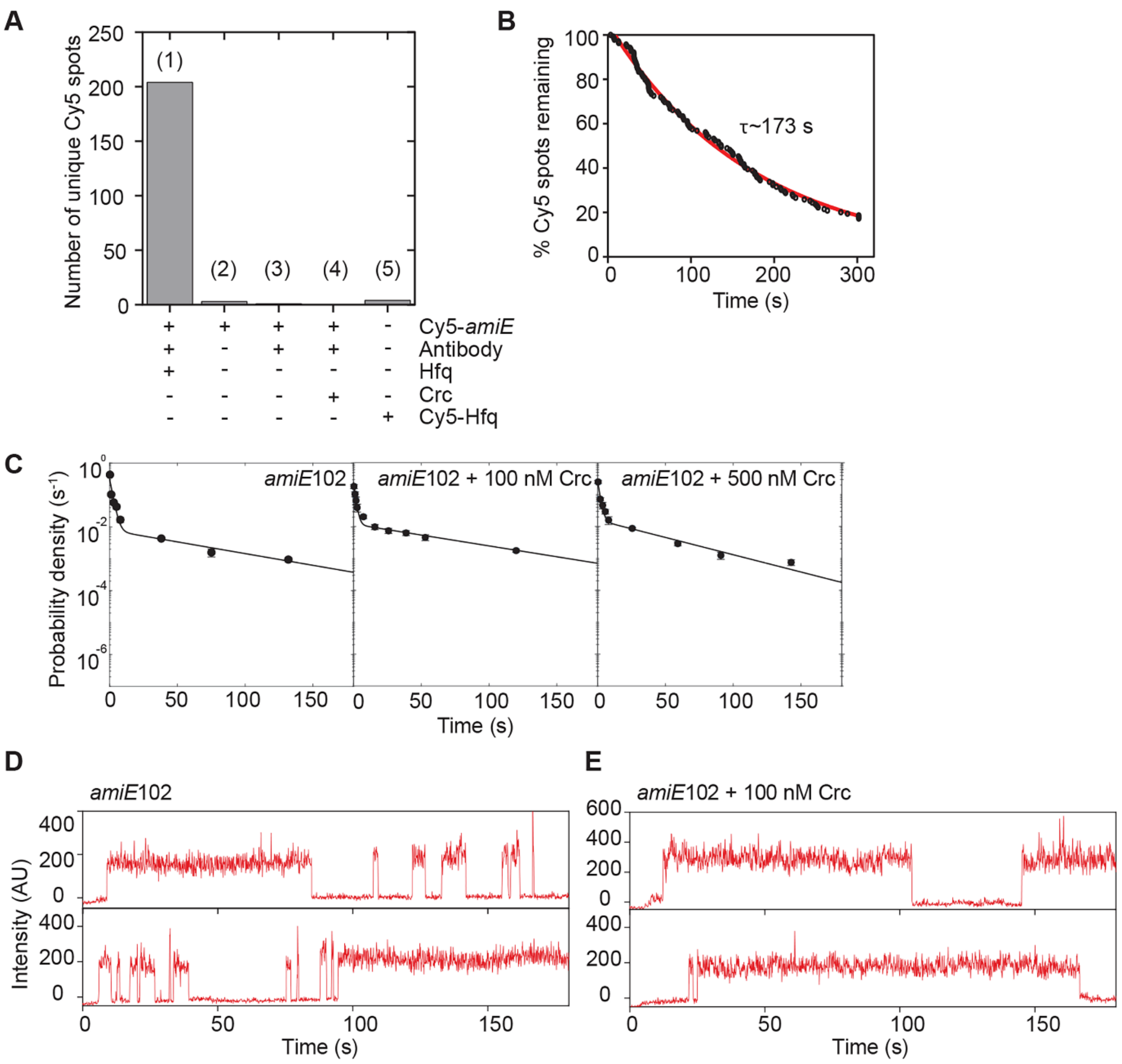

Supplementary Figure S2. Characteriziation of the single-molecule assays visualizing the Hfq/*amiE*102 interactions. (**A**) Number of unique Cy5 spots in the field of view observed for the first 3 minutes upon injection. The experiments shown by bars 1, 3 and 4 were performed on the same slide with the same antibody dilution to minimize variation between the degree of immobilization. Cy5-Hfq* (0.7 dye/hexamer) was used to observe non-specific binding of Hfq to the slide. Hfq was incubated on the slide in the same way as for the experiments with specific immobilization to the antibody. The movie was recorded after washing the slide with imaging

buffer supplemented with glucose oxidase. (**B)** Survival plot of acceptor only (Cy5-labeled DNAs immobilized on the slide) imaged by direct 640 nm laser excitation under standard experimental conditions. Mean acceptor bleaching time was obtained by a single exponential fit to the survival plot. (**C**) Probability density histogram of the lifetimes of Hfq/*amiE*102 bound states, in the absence or presence of Crc. Lines represent maximum likelihood fits to equations with two exponential terms. Error bars represent the standard deviation in a binomial distribution. Details of the number of observations and fit parameters can be found in Supplementary Table S1. (**D, E**) Example of fluorescence traces of Cy5-*amiE*102 binding to immobilized Hfq in the absence (D) and in the presence of 100 nM Crc (E), respectively.

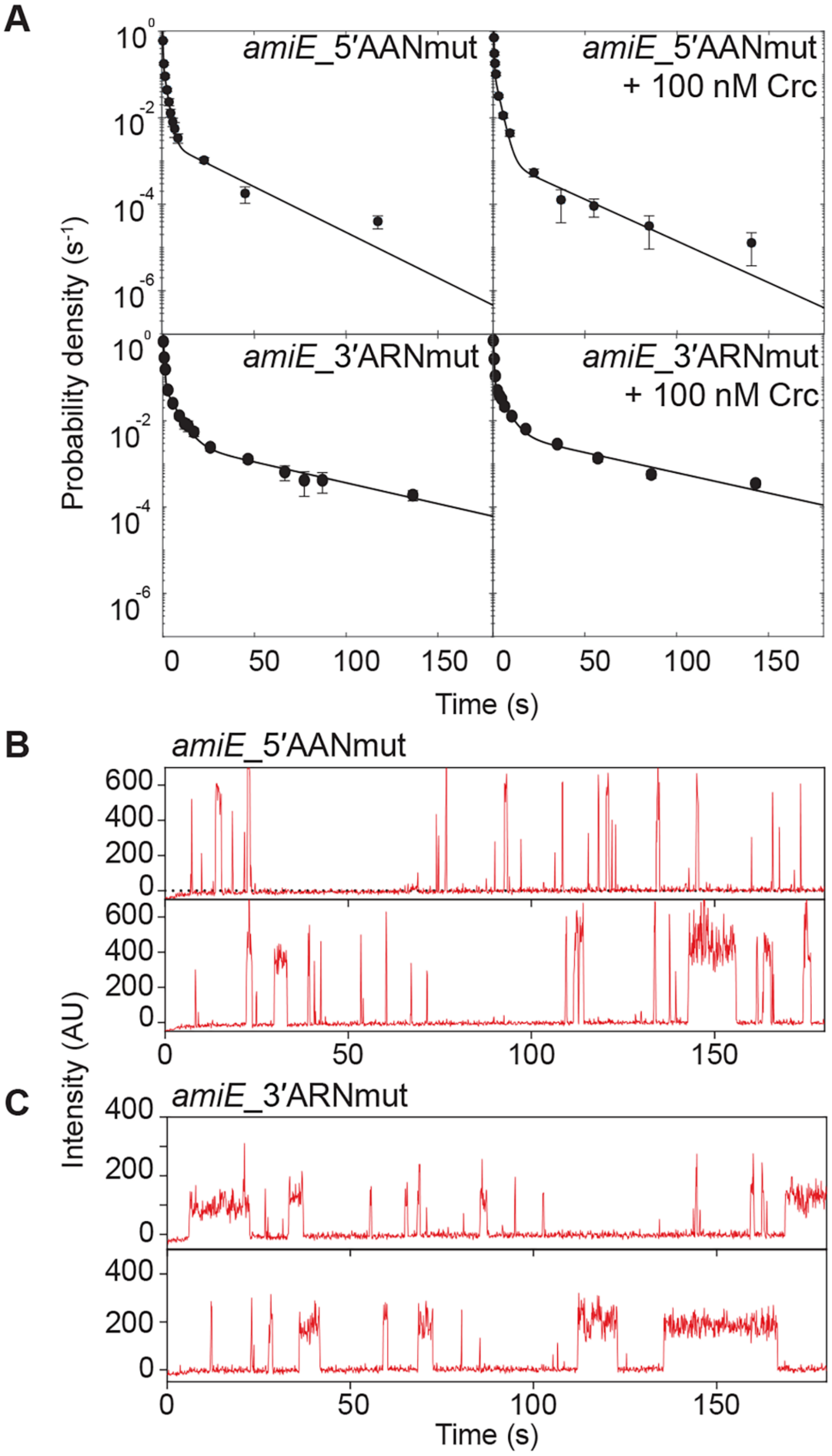

Supplementary Figure S3. Characterization of the single-molecule assays visualizing the interactions between Hfq and *amiE*102 variants. **(A**) Probability density histogram of lifetimes

representing Hfq*/*amiE*_5′AANmut (top panels) and Hfq*/*amiE*_3′ARNmut (bottom panels) interactions, in the absence or presence of Crc. Lines represent maximum likelihood fits to equations with three exponential terms. Error bars represent the standard deviation in a binomial distribution. Details of the number of observations and fit parameters can be found in Supplementary Table S1. (**B, C**) Example of fluorescence traces of immobilized Hfq bound to Cy5-*amiE*_5′AANmut (**B**) and Cy5-*amiE*_3′ARNmut (**C**), respectively.

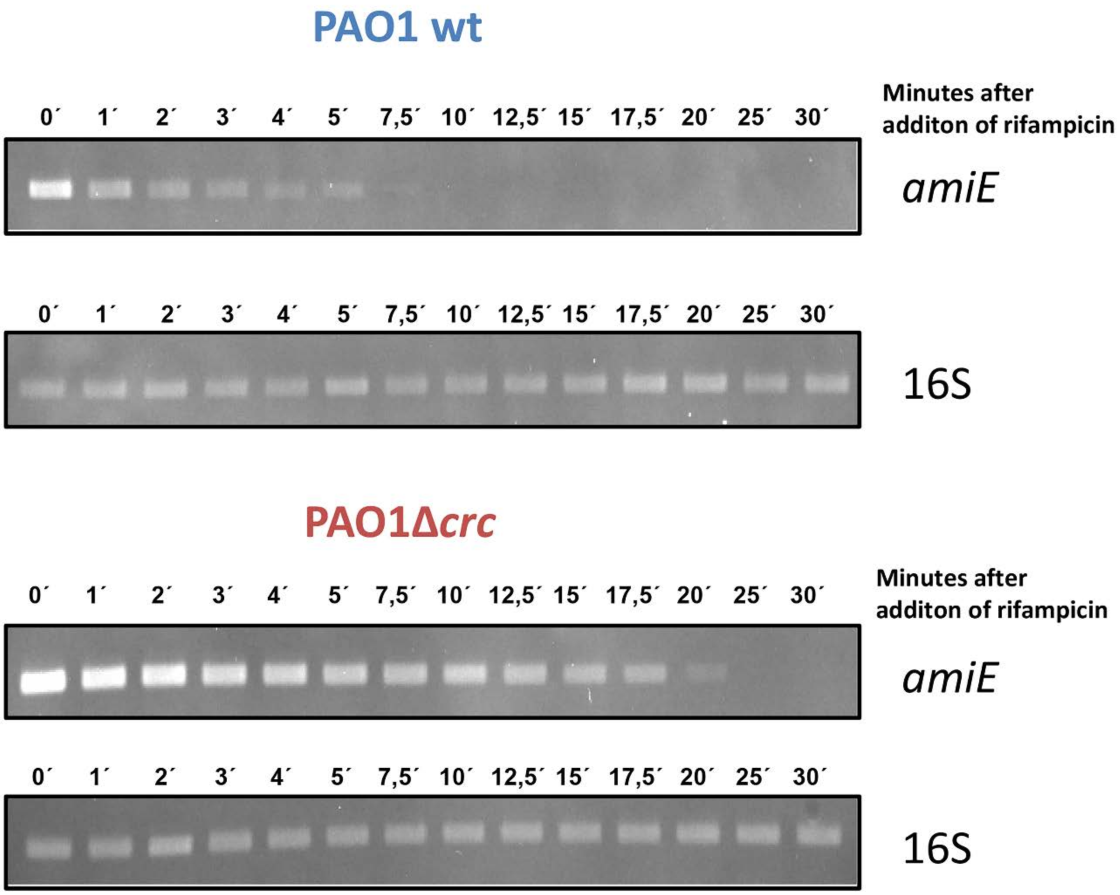

Supplementary Figure S4. Stability of *amiE* mRNA in *Pae* strains PAO1 and PAO1Δ*crc*. PAO1 and PAO1Δ*crc* were grown in BSM medium supplemented with 40 mM acetamide and 40 mM succinate (+ CCR). At an OD_{600} of 1.0, rifampicin was added to a final concentration of 100 µg/ml and samples were withdrawn for total RNA extraction at the times indicated. The remaining levels of *amiE* and 16S rRNA (control) were determined by RT-PCR with oligonucleotides specific for either RNA as described (2). The experiment was performed in triplicate. The result from one representative experiment is shown. The graphical presentation of the experiment is provided in Figure 6.

Experiment	*amiE*, Hfq	*amiE*, Hfq, 100 nM Crc	*amiE*, Hfq, 500 nM Crc	*amiE*_5′AANmut, Hfq	*amiE*_5′AANmut, Hfq, 100 nM Crc	*amiE*_3′ARNmut, Hfq	*amiE*_3′ARNmut, Hfq, 100 nM Crc
Total molecules	108	123	193	116	124	99	183
Total events	440	331	410	1777	1965	843	1153
Binding events/Hfq molecule	4	2.7	2.1	15.3	15.6	8.5	6.3
τ_1 (s) (transient)[a]	-	-	-	0.21 ± 0.01	0.23 ± 0.02	0.45 ± 0.08	0.32 ± 0.05
τ_2 (s) (short)[a]	1.93 ± 0.49	1.35 ± 0.32	1.53 ± 0.62	1.56 ± 0.16	2.2 ± 0.5	4.9 ± 1.3	4.6 ± 1
τ_3 (s) (long)[a]	59 ± 18	64 ± 8.6	40 ± 6	21 ± 3.7	22.5 ± 8.3	45 ± 9.4	46 ± 7.6
a_1 (transient)[a]	-	-	-	0.65 ± 0.03	0.73 ± 0.03	0.54 ± 0.04	0.45 ± 0.03
a_2 (short)[a]	0.56 ± 0.05	0.3 ± 0.04	0.38 ± 0.05	0.3 ± 0.007	0.25 ± 0.006	0.32 ± 0.02	0.32 ± 0.03
a_3 (long)[b]	0.44 ± 0.05[c]	0.7 ± 0.04[c]	0.62 ± 0.05[c]	0.05 ± 0.03[c]	0.02 ± 0.03[c]	0.14 ± 0.05[c]	0.23 ± 0.04[c]

Supplementary Table S1. Fit parameters for the distribution of Hfq/*amiE* binding events under the conditions tested. [a]Parameters from maximum likelihood fits to the binding lifetimes shown in Supplementary Figures S2 and S3. The numbers of molecules analyzed for each condition are indicated. Errors represent the s.d. of the mean determined from bootstrap analysis. [b] $a_3=1-a_1-a_2$, [c] errors obtained by error propagation as described in Materials and Methods.

		Normalized read counts						
**PA number**	**gene**	**PAO1 Δ*hfq***	**PAO1 Δ*hfq***	**PAO1 Δ*hfq*Δ*crc***	**PAO1 Δ*hfq*Δ*crc***	**fold change**	**padj**	**Function**
PA1708	*popB*	1225	1546	3079	3934	2,0	8,8E-04	translocator protein PopB, Type III Secretion
PA1709	*popD*	713	866	1834	2075	2,0	7,5E-04	translocator outer membrane protein PopD precursor, Type III Secretion
PA3476	*rhlI*	440	301	124	90	-2,3	3,5E-04	autoinducer synthesis protein RhlI
PA3785		464	653	138	196	-2,3	1,9E-04	conserved hypothetical protein
PA3973		63	112	243	715	2,3	3,5E-04	probable transcriptional regulator
PA5331	*pyrE*	427	424	165	137	-2,1	5,9E-04	orotate phosphoribosyltransferase
PA5372	*betA*	2542	3066	1030	931	-2,3	2,0E-05	choline dehydrogenase
PA5375	*betT1*	1025	1013	343	246	-2,5	3,8E-06	high affinity choline-specific importer BetT1, functions at low osmolarity

Supplementary Table S2. Transcripts with decreased and increased abundance in PA01Δ*hfq*Δ*crc* *versus* PA01Δ*hfq* grown in LB medium (+CCR) to an OD_{600} of 1.5.
